# The 35th Annual Meeting of the Society of Virology (GfV) in Heidelberg: uniting virology research across German-speaking countries

**DOI:** 10.1128/jvi.01100-26

**Published:** 2026-09-21

**Authors:** Ralf Bartenschlager, Christian Sieben, Stephanie Pfaender, Daniel Sauter, Sandra Ciesek, Hans-Georg Kräusslich

**Affiliations:** 1 Department of Infectious Diseases, Molecular Virology, Medical Faculty Heidelberg, Heidelberg University 152528, Heidelberg, Germany; 2 Nanoscale Infection Biology Group, Helmholtz Centre for Infection Research 28336, Braunschweig, Germany; 3 Research Department Emerging Viruses, Leibniz Institute of Virology 28367, Hamburg, Germany; 4 Institute for Medical Virology and Epidemiology of Viral Diseases, University Hospital Tübingen 27203 , Tübingen, Germany; 5 Institute for Medical Virology, University Hospital, Goethe University 9173, Frankfurt am Main, Germany; 6 Department of Infectious Diseases, Virology, Heidelberg University 9144, Heidelberg, Germany; St Jude Children's Research Hospital, Memphis, Tennessee, USA

**Keywords:** antivirals, cell biology of virus infection, clinical virology, epidemiology, extracellular vesicles, innate and adaptive immunity, structural virology, synthetic immunology, vaccines, veterinary virology, viral vectors, virus evolution, virus-host interactions, zoonoses

## Abstract

The Society of Virology (GfV) is a non-profit association and academic society dedicated to promoting virology across all disciplines in the German-speaking countries of Austria, Germany, and Switzerland. This mission is pursued through a variety of activities, most notably its annual meeting, which provides a stimulating environment—particularly for young scientists and students—to exchange ideas, establish new collaborations, and learn from leading virologists in an informal setting. This year, the GfV held its annual meeting in the picturesque city of Heidelberg, bringing together more than 1,000 participants. The program featured five plenary sessions with distinguished speakers presenting their latest research findings. Topics included virus evolution, the cell biology of viral infection, novel approaches to studying and preventing viral infections with a focus on innovative vaccine strategies, the use of imaging technologies to investigate virus–host interactions, and the development of potent antiviral therapies for chronic viral infections, illustrated by curative therapy of chronic hepatitis C and long-term prevention of HIV-1 infection. The plenary sessions were complemented by 21 parallel workshops covering all areas of virology, as well as several satellite sessions. Many of these were organized by the Young Society of Virology (jGfV), which is dedicated to promoting the next generation of virologists. This report summarizes the presentations delivered by the plenary speakers and provides a brief overview of the activities in the various sessions, including the prize-winning contributions presented at the meeting.

## INTRODUCTION

The Society of Virology (German Gesellschaft für Virologie, GfV) is the leading non-profit academic association for virologists in Germany, Austria, and Switzerland (see Box 1). Founded in 1990, the mission of the GfV is to promote research, education, and interdisciplinary collaboration across all areas of virology, from basic science to clinical and translational applications. Over the past 35 years, with currently more than 1,400 members, the GfV has grown into one of the largest virology societies in Europe, facilitating scientific exchange, supporting early-career researchers, and raising public awareness of viral diseases and their global impact.

Box 1. **History and organization of the GfV**

The Society of Virology (GfV) is the leading non-profit academic association for virologists in Germany, Austria, and Switzerland. Founded in 1990, the GfV was established in response to the growing recognition that virology had developed into an independent scientific discipline that was no longer adequately represented within traditional microbiological societies. From the beginning, the society was conceived as a platform for virologists across German-speaking countries, deliberately bringing together researchers from Germany, Austria, and Switzerland and, at the time, also the former German Democratic Republic.

The society aims to connect basic, clinical, and translational virology, integrating molecular, clinical, veterinary, and environmental perspectives. Early activities were strongly shaped by advances in molecular virology and virus genetics, which later expanded to include immunology and emerging infectious diseases. Over the past decades, the GfV has evolved in parallel with major developments in the field. The increasing relevance of zoonotic infections, the integration of high-throughput sequencing and data-driven approaches, and the growing importance of global health have further strengthened the need for interdisciplinary collaboration. This was particularly evident during the COVID-19 pandemic, which highlighted both the scientific and societal relevance of virology and brought the expertise of GfV members into the public and political sphere. Today, with close to 1,400 members, the GfV represents one of the central virological networks in Europe. It serves as a platform for scientific exchange, supports early-career researchers, and contributes expertise to public health and policy discussions. Its structure reflects the breadth of modern virology and its ongoing role in pandemic preparedness and response. Further information can be found at https://g-f-v.org/en/.

## OVERVIEW OF THE ANNUAL GfV2026 MEETING IN HEIDELBERG

A central event of GfV’s scientific activities is the annual meeting, held each year in a different city in Germany, Austria, or Switzerland. Since 1991, this meeting has brought together virologists from across Europe and beyond. This year, the 35th annual meeting took place in Heidelberg, Germany, in the Life Science Campus of Heidelberg University from 17–20 March. The meeting offered the opportunity to get to know one another, exchange ideas, network, and present their own work, which is particularly important for the next generation of scientists.

**Fig 1:**
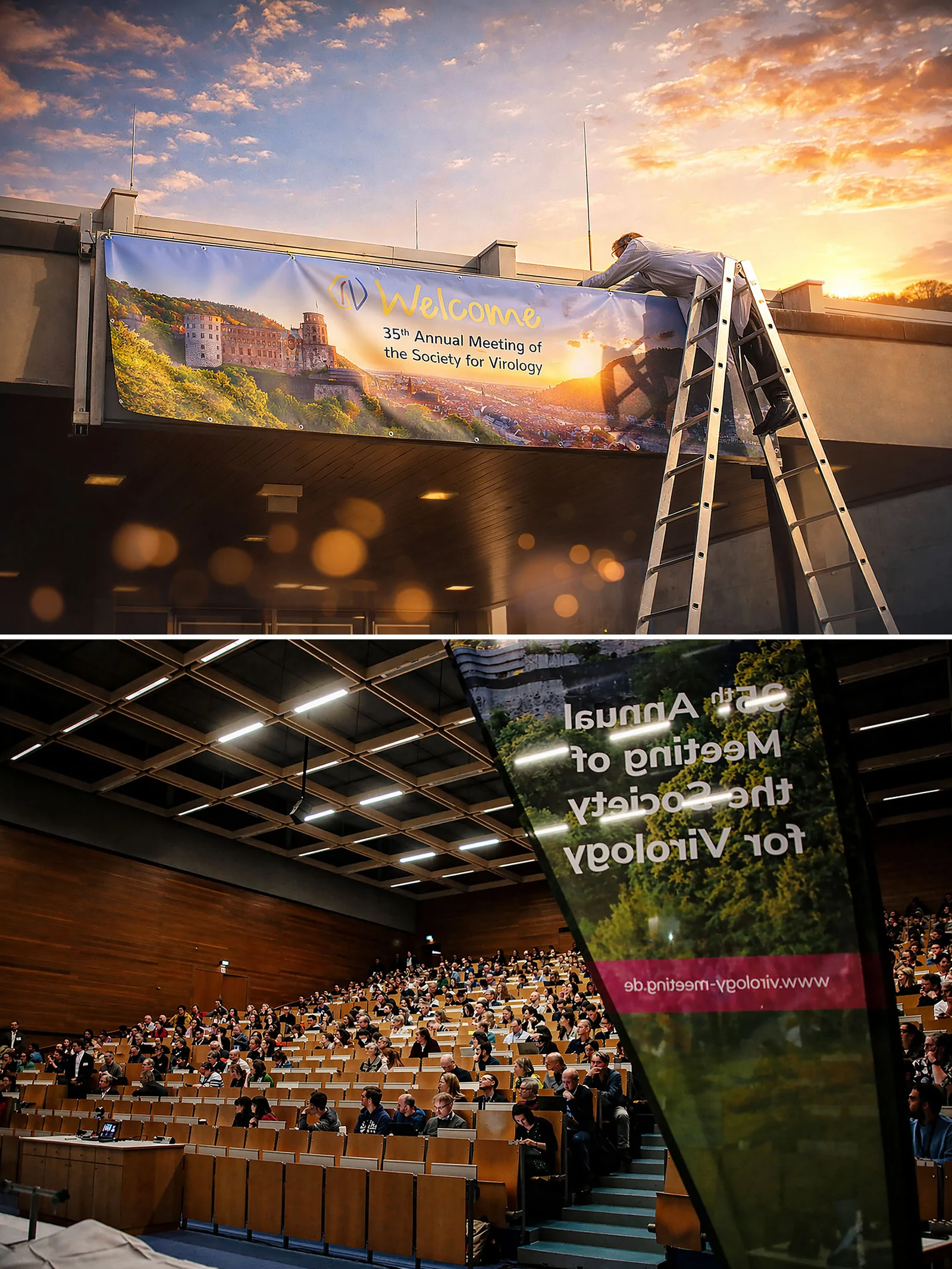
Impressions from the annual GfV meeting 2026 in Heidelberg. (Top) Mounting of the meeting banner on the day before the meeting to guide and welcome the attendees on the next day. (Bottom) A view into the main lecture hall before a plenary session. *©* Claudia Last, published with permission.

With a total of 1,040 participants, the GfV meeting in Heidelberg set a new record. Around 740 abstracts were submitted and presented in 21 workshops and three poster sessions (Fig. 1). The workshops were centered around five plenary sessions and complemented by satellite sessions, several of them organized by the young Society of Virology (German junge GfV; jGfV) (see Box 2) and an award session. An additional award event, the Chica and Heinz Schaller Award for Distinguished Achievements in Virology, was integrated into one of the plenary sessions.

Box 2. **The Young Society of Virology (jGfV)**

Founded in 2021, the jGfV aims to provide a platform and support network for early-career virologists, including PhD candidates and early postdoctoral researchers. A central focus of the jGfV is communication, ensuring rapid dissemination of information on events, funding opportunities, and open positions via regular newsletters, Slack, and social media. In addition, the jGfV organizes an online lecture series featuring international experts and awards prizes to support laboratory visits and recognize outstanding publications. Within its seven study groups, the jGfV offers tailor-made workshops and organizes scientific and networking events for young scientists. Additional offers for career guidance and dedicated mentoring programs create further valuable opportunities for professional development. More information can be found at https://g-f-v.org/en/jgfv/.

## FIVE PLENARY SESSIONS PRESENTING STATE-OF-THE-ART VIROLOGY

### Plenary 1: virus evolution

For the sake of coherence, the series of talks presented in this report differs from the series during the meeting as this and the subsequent plenary session had to be adjusted to the schedules of two of the speakers.

This session revolved around different aspects of virus evolution, and it started with the presentation entitled “From Birds to Humans? The Evolutionary Dynamics of HPAIV H5N1,” given by **Prof. Martin Beer** from the Friedrich-Loeffler Institute in Greifswald, Germany. He first summarized the history of the highly pathogenic avian influenza virus (HPAIV) H5N1. It emerged as a novel clade in domestic waterfowl in China’s Guangdong province in the late 1990s (1), caused sporadic outbreaks in wild and domestic birds between 2006 and 2020, and since then, annual outbreaks with record levels in 2025. This AIV has enormous evolutionary potential and a high number of permissive hosts, in total estimated >10e11 globally, half of them being wild birds. Although transmission of the panzootic clade 2.3.4.4b to humans still is rare, the risk apparently increased in recent years, mainly driven by the global spread of this clade that today comprises ~210 genotypes and reassortants (2). Members of this clade have caused widespread outbreaks across all continents, and owing to its pathogenicity, have enormous impacts on wild birds, biodiversity, and farm animals.

Prof. Beer then raised the question of why genotypes of higher virulence gained epidemiological advantage in wild birds compared to those with reduced virulence. According to his studies, efficient replication in ducks appears superior even with the trade-off of higher virulence (3). Moreover, he elaborated on mammalian spill-over of HPAIV H5 that so far infected >150 different bird and >50 mammalian species (4). Notably, a new host emerged in 2024: H5N1 AIV clade 2.3.4.4b in dairy cattle in the United States. Since then, the virus spread across >1,000 farms in at least 19 U.S. states. While infection seems to be limited to the milking process and infected cow transportation, Prof. Beer pointed out that especially the avian IAV receptor is expressed in udder tissue, explaining why H5N1 AIV can replicate there to extremely high titers. Importantly, the spillover risk of HPAIV H5N1 clade 2.3.4.4b members to humans appears low when the virus remains primarily adapted to birds. Nevertheless, Prof. Beer stressed that due to its evolutionary plasticity, continuous surveillance and sequence analyses are essential, and any further adaptation of HPAIV H5N1 to mammals, including cattle, must be prevented.

The plenary session was continued by **Prof. Johannes Krause** from the Max Planck Institute for Evolutionary Anthropology in Leipzig, Germany. In his talk entitled “Ancient genomes reveal ten millennia of hepatitis B virus evolution,” he presented the first successful reconstruction of complete HBV genomes from prehistoric human remains (5). To overcome low abundance and extensive degradation of viral DNA in these samples, the Krause group combines shotgun sequencing with computational pathogen screening and phylogenetic analysis. In this way, they recovered three complete HBV genomes from two Neolithic and one medieval individual and showed that (i) HBV had circulated continuously in Europe for at least seven millennia and (ii) Neolithic viruses formed highly divergent lineages most closely related to HBV strains infecting African non-human primates. Thus, ancient HBV diversity was most likely substantially higher than today, with entire viral lineages having disappeared during human history.

Prof. Krause then presented a more recent study reconstructing 137 HBV genomes from individuals spanning ~10,500–400 years before present across Eurasia and the Americas (6). This data set enabled reconstruction of HBV evolutionary history over the entire Holocene. Notably, all contemporary human HBV genotypes derive from ancestors existing ~20,000–12,000 years ago and diversifying with human migration and demographic expansion. Surprisingly, the evolutionary dynamics of HBV differ fundamentally from those of many RNA viruses. Rather than exhibiting gradual linear evolution, HBV has experienced repeated lineage replacement, recombination, geographic expansion, and extinction over thousands of years. Prof. Krause concluded that incorporating ancient viral genomes allows calibration of molecular clocks and resolves discrepancies between short-term mutation rates and long-term evolutionary timescales.

In the third talk of this session, entitled “Data Visualization and Data Sharing to Stay Ahead of Changing Viruses,” **Prof. Emma Hodcroft** from the Swiss Tropical and Public Health Institute in Allschwil, Switzerland, reviewed a rather new open-source database called *Pathoplexus*, established by a scientific consortium with important contributions made by herself (7). Although rapid pathogen genome data sharing is one of the most powerful tools to combat infectious disease outbreaks, the COVID-19 pandemic revealed several weaknesses in existing data-sharing systems, including delayed submission of genomic data, inconsistent metadata standards, and concerns about being “scooped.” To overcome these limitations, *Pathoplexus* was established with the philosophy that openness and fair attribution should coexist. Prof. Hodcroft reported characteristics distinguishing *Pathoplexus* from traditional nucleotide sequence archives. These include the open-source infrastructure, the flexible data sharing, the high-quality metadata, powerful search capabilities, and the built-in quality control. In this way, *Pathoplexus* supports not only scientists but also public health agencies, diagnostic laboratories, and governments. Currently, the platform hosts hundreds of thousands of viral genome sequences associated with human infectious diseases and continuously harmonizes, validates, indexes, and provides these data for immediate exploration. Prof. Hodcroft made clear that *Pathoplexus* is not intended to replace but rather to complement established nucleotide sequence archives. As an integrated pathogen knowledge platform, *Pathoplexus* enables more effective genomic surveillance and accelerates research into viral evolution and infectious disease dynamics. It also ensures that contributing researchers and laboratories receive appropriate recognition. Thus, *Pathoplexus* has the potential to increase the speed and quality of global pathogen surveillance and strengthen international collaboration in public health.

### Plenary 2: cell biology of viral infection

Traditionally, the topic virus-host cell interaction receives most abstract submissions, reflecting the strong commitment to this topic by members of the GfV. Therefore, the organizers were pleased to have three international speakers covering a broad range of topics in this area.

The session was opened by **Prof. Wendy Barclay** from the Imperial College in London, U.K., with a talk entitled “Cell factors that determine influenza virus host range.” Addressing determinants of species specificity of AIV polymerase, she elaborated on the host cell protein ANP32A (8). This protein promotes the formation of an asymmetric viral polymerase complex dimer synthesizing genomic and complementary viral RNA. A deletion of 33 aa residues in human ANP32A renders this protein incapable of forming a functional polymerase complex and restricts AIV in human cells. Mutations in the viral polymerase subunit PB2 can compensate for this defect, enabling the virus to utilize human ANP32 proteins and thus, replicate in human cells (8). However, ANP32A is not the only factor interacting with influenza virus (IV) polymerase, and novel cellular interactors have been identified by the Barclay team (9).

Prof. Barclay then described mutations in the H5N1 AIV polymerase contributing to early adaptation to mammalian cells, especially cow cells. While some of these mutations enhance interaction with mammalian ANP32A (10), they do not suffice for full adaptation of H5N1 AIV to humans because of another major restriction: bovine H5N1 IV does not bind to the human receptor, α2-6 sialic acid, and therefore is not transmitted by air between people. However, bovine H5N1 has accumulated mutations around the sialic acid binding site as it circulates in cows. Dr. Barclay’s team has uncovered that a different type of sialic acid receptor, known as NeuGc, is richly expressed in bovine mammary tissue and that bovine H5N1 IV HA is adapting to use this receptor. Humans do not express NeuGc, and so far, mutations in bovine virus HA do not increase binding to human receptors. Nonetheless, owing to its increasing prevalence and ability to replicate inside human cells, there is a risk of adaptation to humans, requiring continuous monitoring. Dr. Barclay summarized that AIV require several mutations across different viral genes in order to adapt to humans and become airborne.

The second presentation, given by **Prof. Nihal Altan-Bonnet** from the National Institute of Health in Bethesda, USA, was entitled “Innate sensor responses set supra-unitary thresholds for successful viral infections.” Starting point of her presentation was an earlier study in her laboratory, showing that enterovirus-infected cells release membranous vesicles containing clusters of virus particles (11). These vesicles deliver virus particles contained therein ‘en bloc’ into a new host cell. This transmission process is more efficient compared with single virus particles, although the latter transmission pathway should reach a higher number of cells. Prof. Altan-Bonnet then discussed several possibilities that might account for higher transmission by en bloc infection (12). Next, she reported an assay combining free virus particle-only containing inoculum with the selective detection of cytoplasmic input viral RNA by *in situ* hybridization. When analyzing inoculated cells a few minutes after viral entry and thus, prior to initiation of RNA replication, she determined a Viral Input Threshold for Replication (VITREP) that is virus- and cell type-dependent. Using various cell and biochemical methods in combination with quantitative mathematical modeling, she showed that multiple viral genomes entering cells suppress the activation of innate immune sensors like RIG-I, MDA-5, and PKR by activating their respective negative regulators, such as DAPK1. Notably, she showed that this process was independent of viral protein synthesis and instead dependent on cytoplasmic viral input quantity. Consistently, infecting cells depleted for these negative regulators required more virus for productive infection. Furthermore, Prof. Altan-Bonnet showed how defective non-replicating viral genomes promote replication: when they enter cells with non-defective but low quantities of viral genomes, they enable the latter to surpass VITREP and replicate.

In the subsequent presentation entitled “How the HUSH complex protects your genome following retroelement invasion,” **Prof. Paul Lehner** from the Cambridge Institute for Therapeutic Immunology and Infectious Disease, U.K., provided an elegant overview of post-integration genome silencing. Only ~1%–2% of the human genome represents protein-coding genes, while ~40% are retroelement-derived, with Long Interspersed Nuclear Element-1 elements (LINE-1s) being the most abundant and active. LINE-1s are repressed by epigenetic mechanisms but can reactivate under conditions such as aging or cancer, and their retrotransposition causes genomic instability and inflammation. A critical executor retaining genome stability and protecting against retroelement invasion is the HUSH complex discovered by the Lehner team (13). This multi-protein complex binds to nascent RNA and acts as an epigenetic repressor, promoting deposition of histone 3 lysine 9 trimethylation (H3K9me3) and inducing local heterochromatin formation. Prof. Lehner then reported that HUSH “recognizes” its target primarily as long single-exon DNA, i.e., DNA lacking introns, a defining hallmark of retroviruses and retrotransposons. Originating from RNA intermediates, these elements must undergo reverse transcription to generate a complementary DNA copy for genomic integration. HUSH also recognizes and silences intronless transgenes introduced into cells, e.g., by plasmid DNA transfection (14). Mechanistically, HUSH binds to nascent RNA and recruits several effector proteins mediating histone methylation (SETDB1) and chromatin remodeling (MORC2). In addition to transcriptionally active LINE-1s and exogenous retroviruses (e.g. HIV), HUSH represses an array of genetic elements and genes also by a noncanonical HUSH silencing pathway (15). Prof. Lehner concluded his talk by raising open questions like the mechanism by which the normal intron-exon structure protects against HUSH-mediated silencing and the role of HUSH in protecting the germline.

### Plenary 3: synthetic and novel approaches in virology

Like many areas within the life sciences, virology has greatly benefited from recent technological advances enabling researchers to address questions that were previously intractable. Among these developments, synthetic biology emerged as a new topic, offering new opportunities especially for vaccinology. Accordingly, this session that was supported by the Hector Fellow Academy (16) covered this topic.

The session was opened by the exciting presentation “DNA origami vaccines elicit antigen-focused germinal centers” by **Prof. Mark Bathe** from the Massachusetts Institute of Technology in Cambridge and Harvard Medical School in Boston, USA. He started by raising the question of how to find a rare antibody-producing cell within a very large immune repertoire. Taking the analogy of hearing a few quietly speaking persons in a crowd where most people are speaking loudly, he explained that the loud people have to be made silent to identify the few quiet ones. Translated into vaccine-induced antibody responses: to induce rare antibodies by vaccination, you have to reduce the induction of competing antibodies targeting, e.g., the immunogen carrier that is often made from proteins that are “loud” to the immune system. A key problem in developing a prophylactic vaccine, e.g., against HIV, is that germline-encoded B-cells having the capacity to develop into producers of broadly neutralizing antibodies (bnAbs) are extremely rare in the human repertoire. Moreover, these B-cells have little natural affinity for the HIV envelope glycoproteins and, therefore, are difficult to be activated by conventional vaccination. Their maturation and expansion require recruitment into the germinal centers of the lymph nodes, where affinity maturation and proliferation occur. Although multivalent display of germline-targeting immunogens on protein nanoparticles can overcome this problem to some extent, these scaffolds themselves induce antibody responses competing with maturation and expansion of rare B cells targeting the desired antigen. To tackle this challenge, the Bathe team developed virus-like particles (VLPs) using DNA origami, i.e., engineered single strands of DNA assembled into virus particle-like structures (17), to serve as carriers for an engineered HIV envelope protein immunogen (18). DNA origami is minimally immunostimulatory, hence “quiet,” and allows precise antigen display via user-designed particle geometry. Prof. Bathe demonstrated that high-density antigen display triggers robust germinal center responses by inducing robust loading with mannose-binding lectin. This, in turn, increases antigen capture by follicular dendritic cells and stronger expansion of antigen-specific B-cells in germinal centers (19). Notably, inclusion of a T-cell helper peptide increased the immune response. Humanized mice immunized with this vaccine or a protein-based nanoparticle vaccine carrying the same epitope showed that DNA-VLPs were superior, especially when the T-cell helper peptide was included (19). Prof. Bathe concluded that reducing off-target responses by means of non-immunogenic DNA-VLPs enhances the priming of bnAbs and may be an attractive approach in vaccinology and active immunotherapies more broadly.

Continuing with approaches from synthetic biology and their application to immunological questions, **Prof. Kerstin Göpfrich** from the Center for Molecular Biology of Heidelberg University, Germany, continued the session with her talk “RNA design: new folds for synthetic immune cells.” As co-spokesperson of the recently established research Cluster of Excellence *SynthImmune* at Heidelberg University, she explained that structural RNA design has traditionally lagged behind DNA nanotechnology and protein engineering, but with the rise of advanced RNA therapeutics, RNA design became the premier frontier in synthetic biology. To overcome the limitations of conventional synthetic immunology that relies on manipulation of pre-existing living cells via genetic engineering (e.g., CAR T-cell therapies), the Göpfrich group uses a “bottom-up” approach (20). It takes advantage of a nanobiotechnological toolbox enabling the construction of highly functional, folded RNA expressed in artificial cellular compartments. Key to success is the group’s own open-source software platform for RNA design (pyFuRNAce) (21) for engineering co-transcriptional RNA origami structures. Prof. Göpfrich presented data on how to integrate membrane-targeting aptamers into cytoskeleton-like RNA nanostructures to control synthetic cell mechanics and function (22). She also showed how mutagenesis can drive emergent phenotypes and functional variance within simple, synthetic droplet systems. Notably, all RNA nanostructures are transcripts from synthetic genes, can be directly expressed inside cells, and plugged into directed evolution pipelines. By merging these RNA nanostructures with targeted ribozymes and aptamers, Prof. Göpfrich aims to engineer synthetic immune effectors. These technologies will provide modular platforms for understanding baseline mechanisms of immune response and drive the next generation of advanced origami vaccines and smart immunotherapeutics.

The last presentation in this session was given by **Prof. Jason McLellan** from the University of Texas at Austin, USA, presenting “Structural studies of bunyavirus glycoprotein complexes.” Hantaviruses, which belong to the order *Bunyavirales*, are enveloped, negative-sense RNA viruses. They can be transmitted zoonotically to humans, who are dead-end hosts, with one exception being the Andes virus (ANDV), which can be transmitted from human to human. A major unresolved question in hantavirus biology has been how the two envelope glycoproteins Gn and Gc assemble into the functional, native spike complex. Previous studies revealed structures of isolated glycoproteins and low-resolution reconstructions of the virion surface, but the molecular architecture of the membrane-embedded Gn-Gc tetramer—the functional unit of viral entry—remained unknown. To address this question, the McLellan team developed an efficient VLP production system suitable for structure determination by cryo-EM (23). He then provided an overview of the ANDV glycoprotein tetramer structure and reported features like molecular interfaces stabilizing the tetramer and the pH sensing mechanism required for acid-induced membrane fusion during viral entry. Prof. McLellan also presented the structure of ANDV-VLPs in complex with a pan-hantavirus Gn–Gc-specific antibody, allowing the identification of epitope-specific interactions. Finally, he elaborated on immunization studies of mice injected with self-replicating RNA encoding wild-type ANDV glycoprotein or variants with VLP-enhancing mutations. Since titers of induced, neutralizing antibodies were comparable, Prof. McLellan concluded that further studies are required to identify the optimal hantavirus vaccine antigen.

### Plenary 4: imaging of virus-host interactions

This session comprised two plenary and one award talk. The first plenary talk was given by **Prof. Christian Eggeling** from the Friedrich-Schiller-University and the Leibniz Institute of Photonic Technology e.V. in Jena, Germany. He started his talk “Observing virus cycling through super-resolution MINFLUX microscopy” by pointing out that virus entry and assembly/budding are very challenging to investigate experimentally. They occur at the nanometer scale, involve highly dynamic interactions, and take place within complex membrane environments. To overcome these hurdles, Prof. Eggeling reported imaging methods making these events experimentally trackable, most notably the combination of super-resolution STED microscopy with fluorescence spectral imaging and fluorescence correlation spectroscopy. He then discussed two examples employing these approaches with great success. First, the demonstration that the plasma membrane is organized into highly dynamic lipid and protein nanodomains providing a mechanistic basis for how HIV-1 exploits transient receptor and lipid organization during virus entry (24). Second, by combining STED microscopy with fluorescence spectroscopy, his laboratory showed that HIV-1 Gag actively remodels the plasma membrane by trapping PI(4, 5)P₂ and cholesterol to create a nanoscale membrane environment promoting efficient virus assembly and budding (25). Prof. Eggeling then moved on to describe MINFLUX nanoscopy (26). He discussed how the substantially higher spatial (2–5 nm) and temporal (sub-millisecond) resolution of MINFLUX enables tracking of individual lipids and viral proteins during HIV assembly (27). This allows direct visualization of molecular interactions that previously could only be inferred from ensemble measurements. Prof. Eggeling concluded that these imaging approaches help to reveal mechanistic principles of virus–host interactions inaccessible to conventional microscopy.

The second talk was given by a distinguished virologist from Sweden representing also the established collaboration of the GfV with the Swedish Society of Virology: **Prof. Anna Overby Wernstedt** from Umeå University. In her presentation “Understanding viral tropism using whole brain imaging and single nuclei RNA sequencing,” she compared the neurotropism of tick-borne encephalitis virus (TBEV) and the closely related, but attenuated and less neurovirulent Langat virus (LGTV).

Prof. Overby first described the recent identification of the TBEV receptor LRP8 and its role in infection of cultured cells and mice (28). To investigate whether receptor expression alone determines TBEV neurotropism, her group generated an LGTV chimera containing the prM and E proteins of TBEV; entry of this chimera is governed by TBEV proteins, replication, and immune evasion by LGTV proteins (29). This chimera retained low pathogenicity and had a brain distribution and neurotropism comparable to LGTV. Thus, viral entry and receptor distribution alone appear to not fully determine tick-borne flavivirus tropism; instead, non-structural viral proteins contribute substantially.

To address how host cellular responses influence viral tropism, Prof. Overby combined Optical Projection Tomography with an *ex vivo* MRI template called OCUM (30), allowing precise co-registration of optical data with anatomical structures and virus-signal quantification (31). She found that LGTV infection in wild-type mice is largely restricted to gray matter regions of sensory systems, whereas in type I interferon receptor (IFNAR) KO mice, LGTV exhibited a much broader tropism. The restricted tropism in wild-type animals was attributed to virus-induced IFN responses, with type I IFN produced by astrocytes and macrophages and type II IFN by NK cells. Notably, in IFNAR KO mice, LGTV tropism shifted from neurons to microglia. This was due to impaired cytokine-induced activation of microglia, rendering these cells more permissive to infection. Prof. Overby concluded that LGTV tropism is strongly shaped by the immune environment and by intercellular communication within the 3D tissue context.

## THE CHICA AND HEINZ SCHALLER FOUNDATION AWARD FOR DISTINGUISHED ACHIEVEMENTS IN VIROLOGY

**Fig 2:**
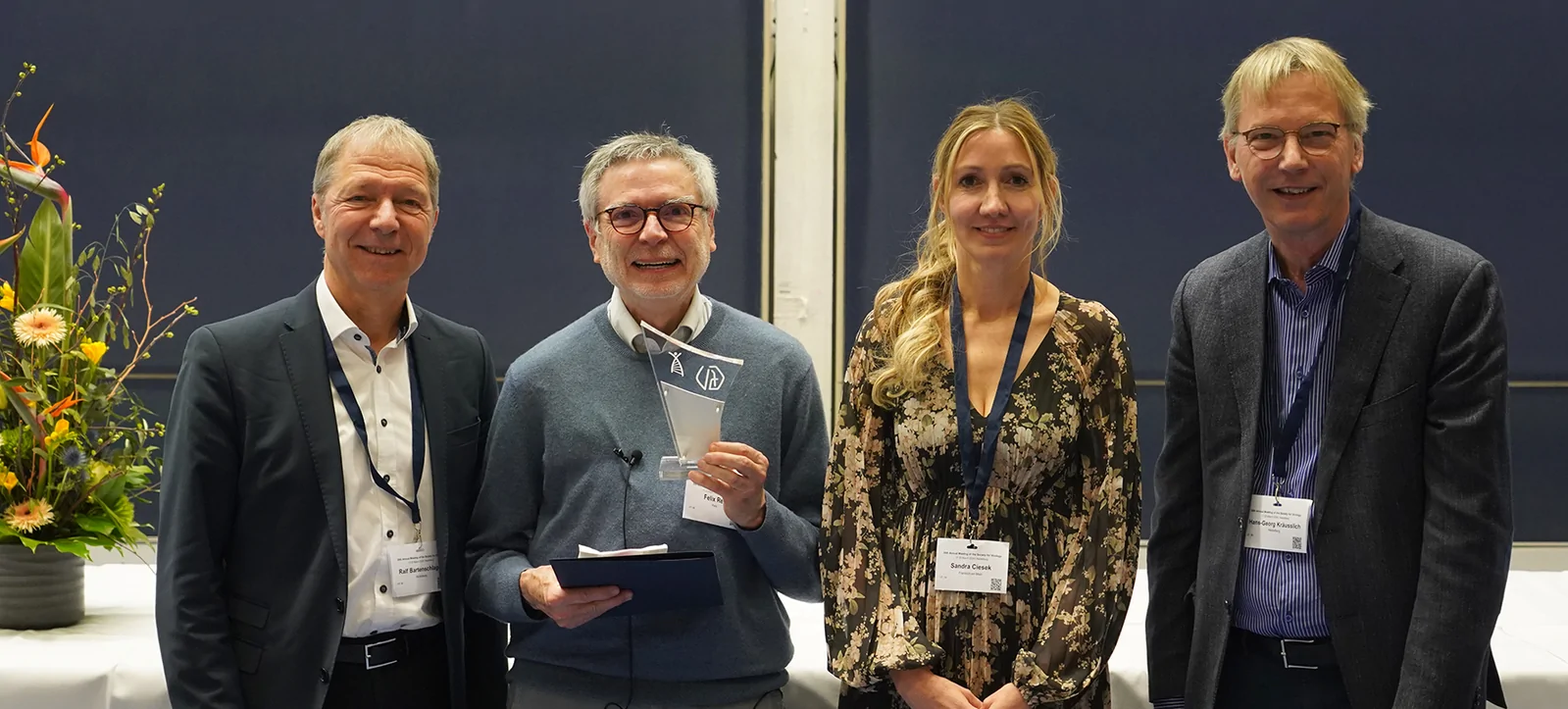
Prof. Felix Rey (second from left) receiving the Chica and Heinz Schaller Award for distinguished achievements in virology from Prof. Ralf Bartenschlager (left), together with the GfV president Prof. Sandra Ciesek and Prof. Hans-Georg Kräusslich, chair of the scientific advisory council of the foundation. *©* Prof. Dr. Volker Lohmann, published with permission.

Since 2022, the GfV has been endowed by the Chica and Heinz Schaller Foundation to establish an Award for Distinguished Achievements in Virology (32). The prize is given to individuals who have made exceptional contributions to the field of virology. It is awarded biennially and endowed with 50,000 Euros. This CHS prize was first awarded to Prof. Galit Alter from Harvard Medical School in Cambridge and in 2024, to Prof. Paul Bieniasz at The Rockefeller University in New York. This year, the CHS award was given to **Prof. Felix Rey** from the Institute Pasteur in Paris, France (Fig. 2). He received the award for his seminal discoveries of the structures of viral envelope proteins, unraveling their fusion mechanisms, ways to neutralize their function by means of antibodies, and for demonstrating that these viral fusion proteins and the eukaryotic gamete fusion protein HAP2 belong to the same structural family and are evolutionarily related. By these means, Prof. Rey has fundamentally shaped modern structural virology and transformed our understanding of the evolution of membrane fusion mechanisms.

In his presentation entitled “The class II membrane fusion machinery in viruses and cells: an evolutionary standpoint,” Prof. Rey first summarized basic features of viral class II fusion proteins using TBEV E-protein as an example as first reported by him (33). He then discussed a major conceptual breakthrough that he and his group made: HAP2 (also termed GCS1), a membrane fusion protein required for gamete fusion in organisms rooted very deeply in the eukaryotic tree of life (metazoans, plants, algae, and protists), possesses the same 3D fold and fusion mechanism as viral class II fusion proteins (34). Thus, the last eukaryotic common ancestor already underwent sexual reproduction using a class II membrane fusion machinery. In the third part of his talk, Prof. Rey described recent studies on an archaeal protein with a class II fusion fold, revealed through metagenomics analyses (35). He presented the development of genetic tools to study an archaeal strain carrying this protein within a mobile genetic element (MGE). Using these tools, he showed that vesicles budding from this strain carry this MGE that is transmitted to other archaeal cells via membrane fusion. Thus, the MGE corresponds to a dsDNA archaeal virus integrated into the genome of its host. Prof. Rey concluded that the class II membrane fusion machinery is evolutionarily ancient and gene exchanges between archaeal viruses and cells have played a central role in the emergence of eukaryotes and sexual reproduction.

### Plenary 5: From molecular virology to clinical success

The last day of the meeting started with a plenary session that was supported by the German Center for Infection Research (DZIF) (36). The session highlighted two prominent examples demonstrating how fundamental research has paved the way for antiviral therapies benefiting millions of patients who suffer from chronic infections with hepatitis C virus (HCV) or HIV.

The first presentation was delivered by Nobel laureate **Prof. Charles M. Rice** from the Rockefeller University, New York, USA. He began his talk, “A cure for hepatitis C, now what?” by drawing parallels between his early work on the establishment of a molecular clone of the yellow fever virus 17D vaccine strain (37), and the challenges the emerging HCV research community was faced with following the publication of the first HCV genome in 1989 (38). Since at that time HCV could not be propagated in cell culture, the field undertook intensive efforts to generate a molecular clone with proven replication competence and infectivity. Prof. Rice described the major roadblocks toward this goal and how his team succeeded in establishing the first HCV genome replicating in intrahepatically inoculated chimpanzees and inducing hepatitis (39). He then outlined the development of the first reliable cell culture systems (40) and their improvements, which laid the foundation for today’s highly effective, all-oral antiviral therapies that can cure chronic hepatitis C in almost all treated patients.

Prof. Rice then turned to the title question “What now?” and outlined two major research directions pursued in his laboratory. The first focuses on establishing a mouse model recapitulating liver disease associated with chronic viral infection. Because HCV cannot be propagated in mice, his group turned to the closely related Norway rat hepacivirus (NrHV) replicating in immunocompetent laboratory mice (41). Importantly, diminishing CD4^+^ T-cell response at the time of infection enabled persistent NrHV infection, closely mimicking chronicity in HCV-infected humans. Persistently infected mice developed chronic hepatitis, progressive liver fibrosis, although not cirrhosis, and eventually hepatocellular carcinoma (42). Thus, NrHV is an experimentally tractable mouse model for studying virus-associated liver disease.

Finally, Prof. Rice presented his recent work on hepatitis B virus (HBV), providing new mechanistic insights into the viral replication cycle (43). He concluded that despite this progress, many fundamental questions in the field of viral hepatitis remain.

An equally exciting talk about another success story was given by **Dr. Stephen Yant** from Gilead Sciences Inc., Foster City, CA, USA. The presentation entitled “Lenacapavir, a new long-acting option for HIV treatment and prevention” started with a brief summary of the discovery of lenacapavir. It was the result of a long-term medicinal chemistry program aiming to identify small molecules targeting the HIV capsid, a viral structure previously considered difficult to drug (44). Playing a pivotal leadership role in bringing this concept to clinical development, Dr. Yant explained that lenacapavir is the first-in-class inhibitor of the HIV-1 capsid protein and a fundamentally new therapeutic principle in antiretroviral therapy. Lenacapavir binds with high affinity to the capsid hexamer interface and disrupts multiple stages of the viral replication cycle (45). This unique mechanism accounts for its exceptional potency and lack of cross-resistance with existing antiretroviral drug classes. As discussed by Dr. Yant, Lenacapavir’s most distinctive feature is its extraordinarily high stability. Initially approved for heavily treatment-experienced patients with multidrug-resistant HIV-1 infection, it has recently demonstrated unprecedented efficacy as pre-exposure prophylaxis (PrEP), reducing HIV acquisition by nearly 100% in large Phase III PURPOSE trials (46). Dr. Yant pointed out that lenacapavir combines a long-lasting, multi-stage antiviral activity with an exceptionally high barrier to resistance, allowing a twice-yearly subcutaneous dosing. These characteristics have the potential to substantially improve both chronic HIV management and global prevention strategies. Dr. Yant concluded that beyond its immediate clinical impact, lenacapavir validates the viral capsid as a druggable target and is expected to stimulate the development of long-acting antivirals directed against structurally conserved components of viral pathogens.

## A SERIES OF 21 WORKSHOPS COVERING THE FULL SPECTRUM OF VIROLOGY

Oral presentations, selected from the more than 740 submitted abstracts, were presented in a total of 21 workshop sessions, each covering six presentations. These addressed a wide range of topics reflecting the breadth of virological research conducted by GfV members. Traditionally, the meeting has a strong focus on basic research, and topics covered in this area included innate and adaptive immunity, pathogenesis and persistence, virus entry and egress, viral replication strategies, structural virology, and virus-host interactions. Regarding the latter, the role of extracellular vesicles (EVs) in viral infection emerged as a new topic that was covered by an extended overview presentation given by **Prof. Esther Nolte-‘t Hoen** from the University of Utrecht, The Netherlands. After explaining the biogenesis and various functions of EVs, as well as their increasing relevance for diagnostics and therapeutic approaches, she described her studies of EVs in picornavirus replication and antiviral defense. Originally described for the hepatitis A virus (47), non-enveloped (naked) viruses more generally can acquire a ‘pseudo-envelope’ with an EV membrane, protecting against neutralizing antibodies and facilitating spread in the infected organism. Earlier studies in the Nolte-‘t Hoen group revealed that cells infected with encephalomyocarditis virus (EMCV) release numerous EV subsets with different compositions and capacities for virus transfer to naïve cells (48) (see also the plenary talk by Prof. Altan-Bonnet). Notably, EV release is induced by the EMCV Leader protein, promoting the accumulation of autophagic structures that are secreted (49). In addition, EV-engulfed virions have an immunomodulatory role by activating monocytes to produce antiviral cytokines. Moreover, naked virus particles can be opsonized and induce FcR-mediated production of inflammatory and antiviral cytokines, whereas virions in EVs cannot. Prof. Nolte-‘t Hoen concluded that EV-enclosed viruses might play a role in viral persistence.

More clinically or epidemiologically oriented virus research was presented in the workshops entitled “Clinical virology,” “Epidemiology and public health,” “Zoonoses and epidemic threats,” and, closely related to that, the workshop “Virus evolution.” Especially the latter has benefited tremendously from new developments in bioinformatics that have revolutionized the field and enable, e.g., the discovery of a large number of hitherto unknown viruses by deep mining of raw sequencing archives.

Both clinical and translational aspects were covered in the workshops “Diagnostics, national reference centers and consulting laboratories,” “Antiviral therapy and resistance,” and the workshop “Viral vectors and vaccines.” Regarding antiviral therapy, new compounds and biologicals targeting a range of different viruses were reported. In addition, several neutralizing antibodies and synergistically acting combinations thereof were presented. The session dealing with viral vectors and vaccines comprised several presentations on influenza virus, including one on membrane-anchored neuraminidase as immunogen, which is less variable than hemagglutinin. Notably, immunization of non-human primates induced broad and cross-reactive humoral immune responses, suggesting that membrane-anchored neuraminidase might have an advantage as a universal influenza vaccine.

Innate and adaptive immunity are important topics. Therefore, two sessions were devoted to “Innate antiviral defenses,” covering presentations on virus sensing by well-known pattern recognition receptors, including the oligoadenylate synthetase (OAS) proteins, but also less studied sensing mechanisms such as the recognition of the HIV envelope glycoprotein by Toll-like receptor 2 (TLR2). Another topic concerned the mechanism of how IFN-induced proteins suppress viral infection, replication, or spread. Regarding adaptive immunity, topics included the correlates of immune protection against MERS-CoV in mice immunized with a measles virus vector encoding the MERS-CoV spike protein or the detection of H5N1 cross-reactive antibodies in two different cohorts of immunized people. Another presentation dealt with the role of Epstein-Barr virus (EBV) in CNS-specific autoimmunity with multiple sclerosis.

## THREE LIVELY POSTER SESSIONS

In line with previous annual GfV meetings, the highest number of abstracts was presented during three poster sessions. Among all the program elements, the poster sessions were the most interactive, offering presenters the chance to enter into direct dialog with the poster viewers. Most posters were presented by PhD and MD students, and they were given the opportunity to get feedback from a large crowd of virologists from diverse fields and with different expertise. As with the other sessions, the aim of the poster sessions was to return home from the meeting with one or two good new ideas for the poster presenter that would move the project forward.

Participants of the meeting could view the posters either during the poster sessions or via the electronic poster viewer at any time during the meeting. Moreover, each participant had the opportunity to vote for their top four posters via an online conference app. In this way, the four winners of the poster prizes that were sponsored by four different journals could be selected without bias. Winners of the poster prizes are listed in Table 1.

**TABLE 1:** Winners of prizes awarded during the annual GfV meeting in Heidelberg

Awardee	Affiliation	Category	Title	Sponsor
Philipp Ralfs	Heidelberg University, Germany	PhD Award 2026	Determinants governing hepatitis C virus negative-strand synthesis and cyto-pathogenesis	Bernhard and Ingrid Fleckenstein Foundation
Katja Dinkelborg	Hannover Medical School, Germany	DZIF Doctoral Award 2026	Identification and characterization of human monoclonal antibodies neutralizing the hepatitis E virus	German Center for Infection Research (DZIF)
Clemens Bodenstein	Robert Koch Institute, Berlin, Germany	Publication Award for Innovative Methods in Virology 2026	Xmas-NT: a novel two-color fluorescence micro-neutralization test for the detection of neutralizing antibodies against monkeypox virus	GfV
Henning Grüll	University of Cologne, Germany	Loeffler-Frosch Award 2026	Development of innovative antibody-based therapies	GfV
Ralf Bartenschlager	Heidelberg University, Germany	Loeffler-Frosch Medal 2026	Plus-strand RNA virus replication: addicted to membranes and vulnerable to drugs	GfV
Felix Rey	Pasteur Institute, Paris, France	CHS-Award for Distinguished Achievements in Virology 2026	The class II membrane fusion machinery in viruses and cells: an evolutionary standpoint	Chica and Heinz Schaller Foundation
Shirin Geravandi	University Hospital Hamburg-Eppendorf, Germany	Poster prize	Changes in antibody levels after vaccination at 6 years of age in HIV-1-exposed uninfected children	*Journal of Virology*
Marie Schwerdtner	Philipps-University Marburg, Germany	Poster prize	The role of host cell proteases in highly pathogenic emerging virus infections—virus activation and beyond	*Journal of General Virology*
Julia Mai	University of Freiburg, Germany	Poster prize	Intrinsic immunity against human adenovirus is achieved by a novel epigenetic silencing complex	*EMBO Journal*
Xiankun Wang	University of Hong Kong, China	Poster prize	Virucidal and endosomal escape inhibition peptide inhibits human adenoviruses in human respiratory organoid and hamsters	*npj Viruses*

## THE GfV AWARDS SESSION

To support virologists and highlight their scientific achievements, the GfV offers a range of awards for different career levels, as well as grants and scholarships (Table 1). For early-career researchers completing their PhD or MD thesis, the GfV presents two Doctoral Awards: one sponsored by DZIF, and the other donated by the Bernhard and Ingrid Fleckenstein Foundation. For postdoctoral researchers, the GfV pre-selects nominees in cooperation with the Robert Koch Foundation, which then awards the Robert Koch Foundation Postdoctoral Award for Virology.

A novel GfV award established in 2025 and given for the first time is the Publication Award for innovative replacement methods in virology. This prize was given in the category “Biosafety, Biosecurity, and Risk Mitigation” to Clemens Bodenstein from the Robert Koch Institute in Berlin for his publication entitled “Xmas‐NT: A Novel Two‐Color Fluorescence Micro Neutralization Test for the Detection of Neutralizing Antibodies Against Monkeypox Virus” (50). This study describes an optimized protocol of a (micro)-neutralization test, reducing assay time by 80% and minimizing the biosafety risk while increasing sensitivity of the read-out.

**Fig 3:**
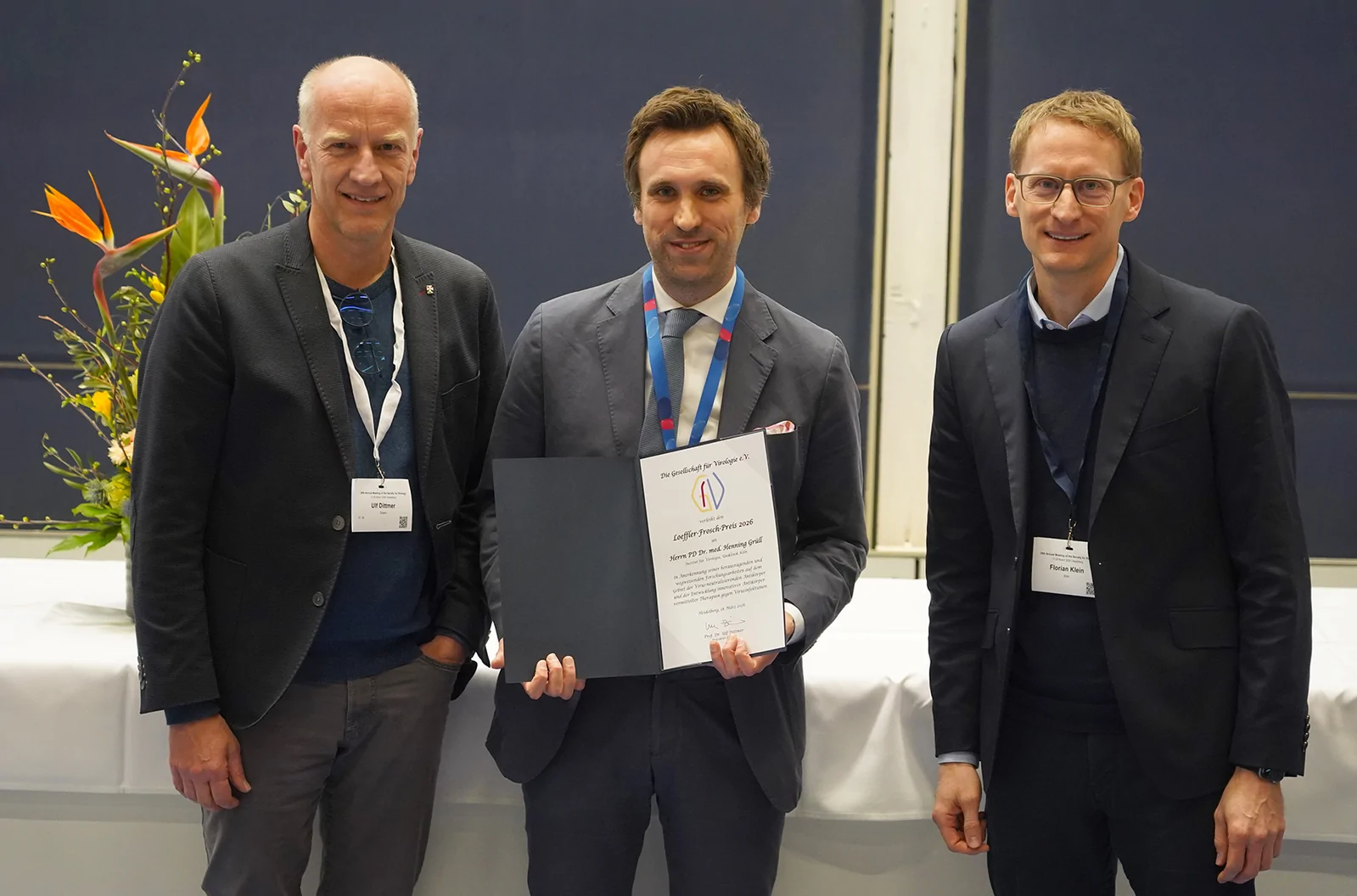
Dr. Henning Grüll (middle), receiving the Loeffler-Frosch Award 2026 of the GfV by past GfV president Prof. Ulf Dittmer (left), together with the laudator Prof. Florian Klein (right). *©* Prof. Dr. Volker Lohmann, published with permission.

The highest GfV prize is the prestigious Loeffler-Frosch prize, named after Friedrich Loeffler (1852–1915) and Paul Frosch (1868–1930), two prominent scientists, who are considered the co-founders of virology and together discovered the first filterable animal virus, the causative agent of foot-and-mouth disease, in 1898. Established in 1994, the Loeffler-Frosch prize is awarded annually for outstanding achievements in virology by young researchers who do not yet hold a lifetime professorship. This year, the prize was given to Dr. Henning Grüll from the University of Cologne (Fig. 3) for his major contributions to decipher human antiviral immunity and to use gained knowledge for the development of innovative antibody-based therapies.

As clinician scientist, Dr. Grüll investigated the first-in-human use of new, highly potent HIV antibodies (51) and discovered new therapeutic principles, including the use of antibody combinations in viremic (52) and pretreated individuals living with HIV (53). He was also among the first to investigate new antibody-mediated strategies in humans aimed at achieving a functional cure for HIV infection (54). For instance, Dr. Grüll identified a novel, highly active HIV neutralizing antibody that prevents antiviral resistance and has recently entered into clinical trials (55). He also studies different applications of neutralizing antibodies, e.g., via inhalation to treat SARS-CoV-2 infection, or by means of antibody-encoding RNA or stable expression via AAV-based vectors. Notably, expression of HIV-1 bNAbs via RNA- and AAV-based approaches effectively suppressed viremia in humanized mice. In the last part of his talk, Dr. Grüll showed an amazing example of how antibody avidity can be made use of to overcome reductions in neutralizing potency.

To honor lifetime achievements in virology in the German-speaking region, the GfV awards the Loeffler-Frosch medal to distinguished virologists close to the end or after their active careers. This year, the medal was given to Prof. Ralf Bartenschlager from Heidelberg University for his major contributions to the study of HCV and orthoflaviviruses and his efforts to promote young scientists. In his presentation, he reviewed the biogenesis, architecture, and composition of the membranous replication organelles induced in cells infected with HCV or orthoflaviviruses (56). He also described the mode-of-action of antiviral drugs targeting these organelles: clinically used NS5A inhibitors of HCV and an NS4B inhibitor targeting DENV (57). Initially considered to be non-druggable, both proteins turned out to be valid drug targets, and in the case of HCV, NS5A inhibitors became a cornerstone of curative therapy. Prof. Bartenschlager concluded that targeting viral replication organelles is an attractive approach for antiviral therapy.

## THE YOUNG SOCIETY OF VIROLOGY (jGfV): PROMOTING THE NEXT GENERATION OF VIROLOGISTS

At this year’s annual GfV meeting in Heidelberg, the jGfV organized several activities to foster exchange between early-career scientists (ECS) and established researchers. A highlight was a lunch discussion with several invited speakers (Profs. Charles Rice, Mark Bathe, Anna Överby, Nihal Altan-Bonnet) and an EMBO Journal editor (Ieva Gailite). The discussion was very well received, with highly positive feedback from both students and invited speakers. As an additional activity, the jGfV organized a “Sticker Quest,” in which participating PIs were represented by collectible stickers and identifiable via white lanyards, encouraging informal interactions with ECS. A clear highlight was a dedicated session designed by the jGfV student representatives 2025/2026, Lisann Röpke and Sophie Marie Aicher. Following a brief introduction to jGfV activities by the jGfV speakers, Christian Sieben and Stephanie Pfänder, they hosted an interactive quiz entitled “Pint of Virology,” which combined entertainment with scientific learning and attracted a large audience. The session concluded with the farewell of the outgoing student representatives and presentations from candidates for the new positions. Overall, the meeting was highly successful and provided a stimulating platform for networking, exchange, and engagement within the jGfV community.

## CONCLUSIONS

The GfV meeting 2026 once again highlighted the strength and diversity of virology research in German-speaking countries. Outstanding presentations covering a broad range of topics showcased the high quality of science and provided exciting insights into current developments in the field. The meeting also offered excellent opportunities for young scientists to present their work, interact with leading experts, and build professional networks. Beyond the scientific program, the conference fostered lively discussions, new collaborations, and valuable personal interactions. The stimulating atmosphere and enthusiastic participation made the meeting both scientifically rewarding and highly enjoyable, ensuring that the Heidelberg meeting will be remembered as a successful and rewarding gathering for the virology community. Building on this success, the virology community now looks forward to reconvening at the next annual GfV meeting, which will be held in Frankfurt from 1 to 4 March 2027.
